# Single-pixel imaging using caustic patterns

**DOI:** 10.1038/s41598-020-59224-8

**Published:** 2020-02-10

**Authors:** Ermes Toninelli, Daan Stellinga, Bereneice Sephton, Andrew Forbes, Miles J. Padgett

**Affiliations:** 1 School of Physics and Astronomy, University of Glasgow, Glasgow, G12 8QQ UK; 2 School of Physics, University of the Witwatersrand, Johannesburg, South Africa

**Keywords:** Acoustics, Imaging techniques

## Abstract

Single-pixel imaging uses a time-varying transmission mask placed in the illumination to achieve imaging without the use of detector arrays. While most research in this field uses sophisticated masks implemented using spatial light modulators, such methods are not available at all lengthscales and wavelengths of illumination. Here we show that alternatively a sequence of projected caustic intensity patterns can be used as the basis for the single-pixel imaging of objects. Caustics can be formed using slowly varying random phase masks, such as for example the surface of a swimming pool, which potentially makes using caustics an option at a range of lengthscales and wavelengths.

## Introduction

Single-pixel cameras are a form of computational imaging where a time-varying mask is used to create projected patterns to illuminate an object and a single-pixel detector used to measure the total time-varying intensity of the back-scattered light. Equivalently, the object can be illuminated full-field and the time varying mask inserted prior to the detector. In both configurations, the signals from the detector are a measure of the overlap between the mask pattern with the object and, as pioneered by Baraniuk and co-workers, given many such measurements corresponding to different patterns it is possible to reconstruct an image of the object^1,2^. One advantage of such single-pixel approaches is that they remove the need for detector arrays, which can be prohibitively expensive, or simply unavailable, at some operating wavelengths.

Much of the research in the area of single-pixel cameras has been directed towards the choice/design of mask patterns and the algorithms needed to reconstruct the image from these patterns and detector data^3^. The choice of patterns ranges from patterns based upon random laser speckle^4^, to those implemented using spatial light modulators capable of creating complicated masks at very high refresh rates. These possible masks include both Hadamard^5^ and more bespoke patterns aimed at optimising specific scenarios^6^. However, the use of a spatial light modulator sets a limitation upon the operating wavelengths, requiring sophisticated solutions at any wavelength much removed from the visible spectrum^7^.

Rather than using designed masks, the use of laser speckle makes possible the application of a wide variety of random processes for the generation of the illumination patterns, but still leaves the problem of needing to know the precise spatial form of the patterns produced^8,9^. Measuring these patterns with a detector array or other imaging system would largely negate the operational advantage of the single-pixel approach, hence the form of the individual patterns need to be predicted from knowledge of the process producing it. This prediction might be direct from knowledge of a transmission mask imaged to the plane of the object, or from knowledge of the mask and the subsequent computational propagation of the light to the object plane. As an alternative to speckle, which can be difficult to predict, in this work we consider the use of caustic patterns that are generated whenever light is reflected or transmitted through a slowly varying phase mask^10^ (for example the patterns formed on the bottom of a swimming pool, resulting from the imperfect focusing of the sunlight by the ripples on the surface of the water). Although these slowly varying phase masks still produce random speckle patterns in the far-field, predictable caustics are produced at a characteristic propagation distance, with the transition between the two regimes having been studied previously^11^. Using caustics for single-pixel imaging has also been postulated previously, but in that work did not yield recognisable images^12^.

In this work, we perform a proof of principle single-pixel imaging using caustic patterns, which although we create using a programmable spatial light modulator (SLM), at other wavelengths could be created with other systems. One possible alternative system would be the use of deformable mirrors which are reflective over an extended wavelength range and can create smoothly varying height profiles that after propagation will give rise to caustics in the reflected light.

The use of projected patterns for single-pixel imaging should not be confused with the use of projected patterns in structured light microscopy, the latter combines structured illumination (including speckle) with conventional spatially resolved imaging to achieve sub Rayleigh resolution^13^. By contrast here we use projected patterns simply to remove the need for a detector array.

## Results and Analysis

**Figure 1:**
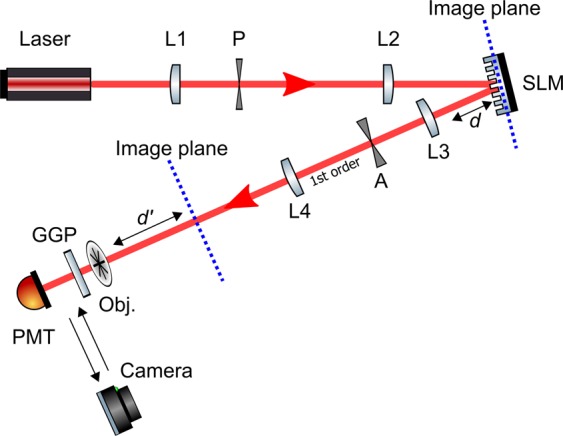
Experimental realisation. The output from a HeNe laser is magnified and spatially filtered using a 50 mm focal length lens (L1), a 50 *μ*m precision pin-hole (P) and a 400 mm focal length lens (L2). A Cambrige Correlators liquid-crystals SLM (model: SDE1024) is used to impart the caustics hologram onto the incoming collimated beam. The first diffracted order is selected by an aperture (A) placed in the far-field of a 250 mm focal length lens (L3). A 500 mm focal length lens (L4) is used in conjunction to L3 to magnify the caustic patterns. The plane of the SLM and the plane of the virtual modulator are highlighted by the blue-dotted lines. A ground-glass plate (GGP) is used to scatter the light ensuring that the single-pixel detector (Thorlabs photo-multiplier tube (PMT), model PMM01) collects light from all regions of the object. The object (Obj.) can be placed at some distance $d$ from the image plane of the SLM, corresponding to a distance $d{\prime}$ from the magnified plane of the virtual modulator. In order to verify that the experimentally generated caustic patterns matched the modelled ones, a camera (Canon EOS 5D Mark III) was placed with its sensor array directly in the plane of the object.

As shown in the experimental arrangement in Fig. 1, the output beam from a HeNe laser is collimated and expanded to fill the aperture of a spatial light modulator. This modulator is programmed to display a smoothly varying random phase mask that is fully within the $0-2\pi$ range. The scale of these phase fluctuations is such that the light is approximately focused to a few 100 mm after the plane of the modulator (c.f. the bottom of a swimming pool). To ensure the fidelity of the phase fluctuations, the modulator is used in diffractive mode where the addition of a phase-grating to the phase mask produces the desired complex beam in the first diffracted order, which is selected in the far-field of the modulator using an aperture, and then re-imaged to create a virtual modulator at some other plane. The object is then placed at the required distance after the plane of this virtual modulator. The overlap between the object and the caustic pattern is measured in transmission using a large area photomultiplier. A ground-glass diffuser (1500 grit) inserted between the object and the detector prevents the finite diameter of the detector vignetting the image. The objects used in this publication consist of 10 mm × 10 mm laser printed acetate sheets.

**Figure 2:**
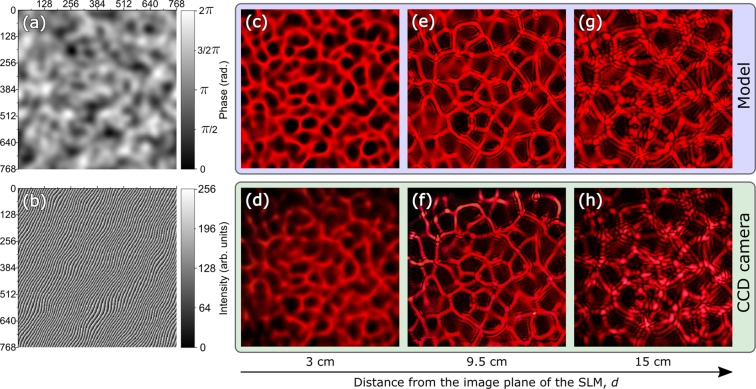
Generated caustic patterns using a liquid-crystals SLM. A phase mask (**a**) is used to generate a hologram (**b**) that is displayed on the SLM. A numerical model is used to model the intensity patterns at three increasing distances from the image plane of the SLM (3, 9.5 and 15 cm) showing a transition from caustic to speckle. The modelled intensity patterns are shown in (**c**,**e**,**g**) whereas the intensity patterns, as recorded by a camera, are shown in (**d**,**f**,**h**). The distance *d* between the camera plane and the plane of the SLM for each pattern is reported at the bottom.

The spatial form of the projected caustic patterns at some distance from the plane of the SLM can be modelled from the knowledge of the phase masks displayed on the SLM using plane-wave decomposition. Additionally, it is possible to verify that the measured caustic patterns are the same as the modelled ones, by replacing the object with a focal plane array (we used a lensless Canon EOS 5D Mark III, due to its large chip size). The random phase mask is calculated by assigning every pixel a random value and then applying a spatial low-pass filter to obtain a slowly varying random pattern which can then be rescaled to lie in the $0-2\pi$ range and displayed on the modulator. Figure 2 shows examples of the phase masks applied (a), the corresponding phase mask displayed on the SLM with an added grating (b). The distance *d* from the plane of the SLM corresponds to the distance *d*′ from the magnified plane of the virtual modulator.

The scale and depth of the phase fluctuations were chosen such that high contrast caustics were formed at a distance approximately $d{\prime} =9.5$ cm from the magnified image plane of the SLM. Knowing the spatial form of the phase fluctuations allows the accurate structure of the caustics at arbitrary distances from the plane of the virtual modulator to be predicted, as shown for three distances in Fig. 2(c,e,g). Corresponding caustics measured by means of a camera at the same distances are shown in (d,f,h). We note that the agreement between the modelled and measured caustic patterns is high. Moreover, as the distance is increased beyond the designed 9.5 cm, the spatial form of the projected caustics tends towards a speckle pattern, as visible in (g,h).

Using a sequence of $i=\mathrm{1..}.N$ random caustic patterns to illuminate the object and measuring the single-pixel signal allows the image to be reconstructed using standard single-pixel camera algorithms. If the caustic patterns are described by ${p}_{i}(x,y)$ and the corresponding measured signal from the single-pixel detector are ${s}_{i}$ then a traditional reconstruction algorithm for the image $I$ from $N$ mask patterns is^8,14^:

1 $$I=\mathop{\sum }\limits_{i=1}^{N}({s}_{i}-\langle {s}_{i}\rangle ){p}_{i}(x,y)$$

where $\langle {s}_{i}\rangle$ is the average value of all the single-pixel detector signals.

In this work we have not used complicated image reconstruction protocols or image post-processing, since we aim to establish the basic principles from which recognisable images are obtained without recourse to sophisticated denoising algorithms or use of image priors. The only alteration made to the basic algorithm described in Eq. 1 is limiting the average signal $\langle {s}_{i}\rangle$ to only include the most recent 100 measurements, so as to reduce the effect of detector drift on the reconstruction.

**Figure 3:**
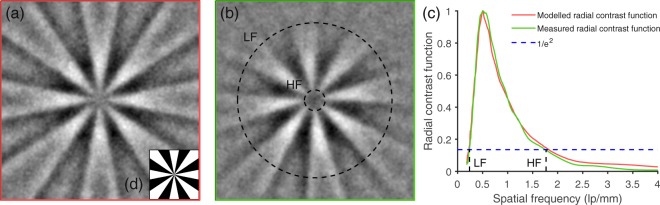
Radial contrast functions. Images of a Siemens resolution-target (shown in the inset (**d**)) were reconstructed using 30,000 modelled patterns and modelled signals (as shown in (**a**)), and using the same 30,000 modelled patterns with measured signals (as shown in (**b**)), both at a resolution of 768 × 768 pixels. The corresponding radial contrast functions are shown by the red and green series in (**c**). The contrast functions highlight the lack of low frequency information also apparent in the images due to the lack of low frequencies inherent to caustic intensity patterns. The low and high spatial frequencies are marked at the $1/{e}^{2}$ amplitude shown by the dotted blue-series and labelled LF and HF respectively.

Images of an eight-spoke Siemens resolution-target reconstructed using the aforementioned algorithm are shown in Fig. 3. The images shown in (a) were created using a simulated perfect setup, with the signals calculated from overlap between a perfect representation of the target (*o*) and the predicted caustic patterns at 9.5 cm, i.e. $${s}_{i}= < {o}|{p}_{i}(x,y) > $$. In (b) the actual experimentally measured PMT values are used alongside the same predicted caustics. As anticipated the two reconstructed images are highly similar, confirming the accuracy of the modelled patterns. Caustic patterns modelled at 9.5 cm from the image plane of the SLM, such as those shown in Fig. 2(e), were used for these reconstructions.

The radial contrast functions (red- and green-series) shown in Fig. 3(c) were extracted from the pixel intensity values of the reconstructed images of the Siemens-star resolution target shown in Fig. 3(a,b). The low and high spatial-frequency limits (labelled LF and HF respectively) were defined using the $1/{e}^{2}$ criterion as applied to radial contrast. The images obtained appear to lack the low frequency information present when imaging using other means, resulting in an edge enhancement effect to the images.

**Figure 4:**
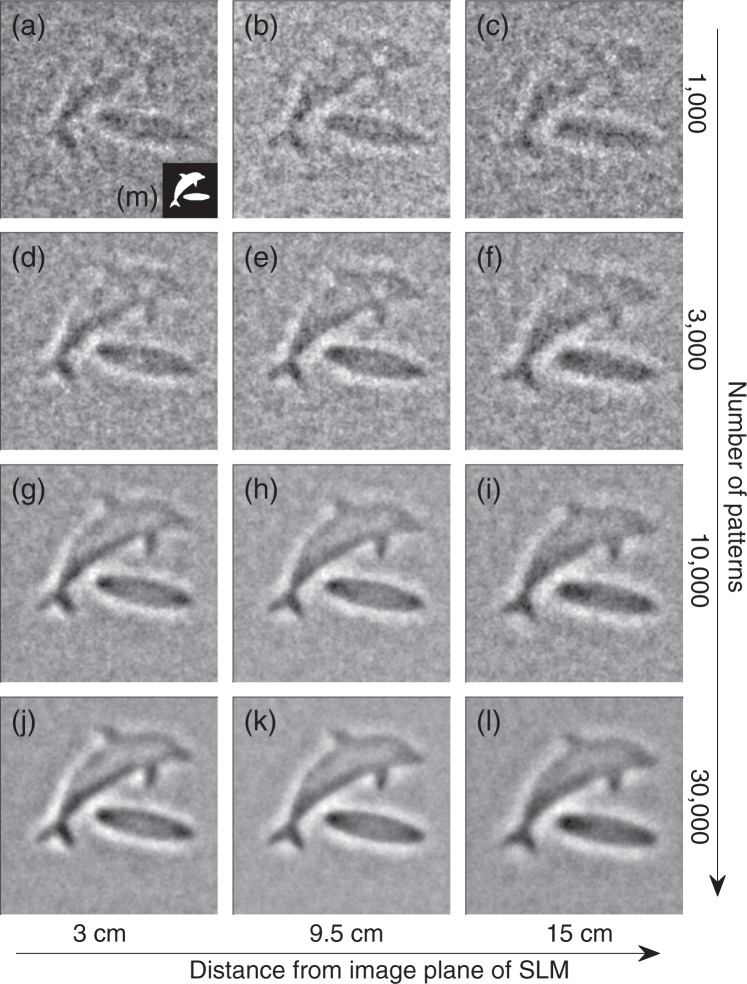
Reconstructed images with a 768 × 768 resolution of a general binary object using caustic patterns. The object shown in (m) was reconstructed using the measured PMT signals when the object was illuminated with a set of caustic patterns. The reconstructions shown were performed with the object placed at various distances beyond the image plane of the SLM (3 cm for **a,d,g,j**; 9.5 cm for **b**,**e,h,k**; and 15 cm for **c**,**f**,**i**,**l**), and with various numbers of caustic patterns included in the reconstruction (1,000 for **a**–**c**; 3,000 for **d**–**f**; 10,000 for **g**,**i**; and 30,000 for **j**–**l**). In all cases the caustic patterns used in the reconstruction were taken from the same set and calculated for an expected distance of 9.5 cm.

This edge enhancement effect is also clearly visible in the images shown in Fig. 4. The images of a binary amplitude object in the shape of a dolphin (shown in (m)) were all acquired experimentally using the same methods as the Siemens star in Fig. 3. The rows in Fig. 4 show the effect of increasing numbers of patterns used in the reconstruction, while the columns show the effect of placing the object at various distances from the image plane of the SLM. Note that the caustics patterns used for the reconstructions in this figure are all identically calculated for a distance of 9.5 cm, so this is a measure of the depth of field of the system.

While as expected more patterns leads to better images, binary images of this type are already recognisable at 1,000 patterns, and reasonably well defined with as few as 3,000 measurements, especially at the target distance. From 10,000 to 30,000 patterns the outlines of the image no longer significantly improve, though the noise in the contiguous areas of the image is clearly reduced further. The figure also shows that the object distance from the modulator is not crucial, with convincing images reconstructed at all three distances shown, even though only the middle column uses the patterns actually projected on the object in the reconstruction, as shown in Fig. 2(f). The left and right most columns will instead have been illuminated by patterns like those shown in Fig. 2(d,h) respectively, but this only appears in the reconstructions as an apparent slight defocus.

## Conclusions

In this work we have shown that a sequence of projected caustic intensity patterns can be used as the basis for single-pixel imaging of objects, albeit images which are inherently edge-enhanced. Since caustic patterns are a natural consequence of smoothly varying random phase masks, the approach could be applied to a wide range of possible wavelengths, wave types, and various implementations of phase masks. For example, caustics are formed by the refraction of light by ocean waves and hence could be used to perform imaging of submerged objects. However, note that in this case the surface profile of the water would also have to be known so that the spatial form of the resulting caustics could be calculated and used within the reconstruction algorithm.

The data in support of this publication (i.e. the measured signals from the PMT and a caustic patterns generator) is available at the following repository^15^.
